# Comparison of Amplix® hepatitis B virus and Roche COBAS AmpliPrep/COBAS Taqman hepatitis B virus assay in quantifying HBV DNA in plasma of chronic hepatitis B in Senegal

**DOI:** 10.11604/pamj.2021.38.294.24819

**Published:** 2021-03-22

**Authors:** Gora Lô, Anna Julienne Selbé Ndiaye, Amina Sow, Amina Fall, Aissatou Sow, Oumar Barry, Ndèye Marième Diop, Halimatou Diop Ndiaye, Ndèye Coumba Touré Kâne, Souleymane Mboup

**Affiliations:** 1 Centre Médical Inter Armées Lemonier, Dakar, Sénégal,; 2 Institut de Recherche en Santé, de Surveillance Epidémiologique et de Formation, Dakar, Sénégal,; 3 Laboratoire de Bactériologie Virologie de l´Hôpital Aristide Le Dantec, Dakar, Sénégal,; 4 Hôpital Dalal Jaam, Dakar, Sénégal

**Keywords:** Amplix®, hepatitis B virus, Roche COBAS AmpliPrep/COBAS Taqman, deoxyribonucleic acid

## Abstract

**Introduction:**

quantification of hepatitis B virus DNA, a key element in the management of chronic hepatitis B, allows a more direct and reliable measurement of viral replication and monitoring of the virological response to therapy. Polymerase chain reaction (PCR) platforms performing this quantification and adaptable to intermediate laboratories have been developed. Thus, this study was conducted to evaluate the on-site performance of the AMPLIX® hepatitis B virus (HBV) real-time PCR technique in comparison with the COBAS AmpliPrep™ technique.

**Methods:**

performance of the AMPLIX® HBV real-time PCR technique was evaluated with repeatability and intermediate precision (reproducibility) determined. The comparison with COBAS Taqman was performed by testing, in parallel, 42 plasma samples. The statistical analysis using Meth Val® software was focused on correlation and concordance determination.

**Results:**

AMPLIX® real-time PCR assay showed good reproducibility for the low (CV=6.65%) and high (CV=3.15%) control levels but also good repeatability for both the low (CV=2.12%) and high (CV=1.60%) concentration levels. Accuracy obtained in our study were less than acceptability limit fixed to 5%. Viral load measurements between Amplix and COBAS Taqman correlated strongly with a correlation coefficient of 0.97%. Concordance analysis gave an average of the differences of 0.54 log IU/L between the viral load measurements of the 2 techniques.

**Conclusion:**

based on these results, the Amplix real-time PCR platform for the quantification of HBV DNA can be considered as a reliable system for the monitoring of chronic hepatitis B and also a system adapted to intermediate laboratories.

## Introduction

Chronic hepatitis B is a major global health problem, affecting more than 240 million people worldwide and leading to one million deaths each year [1]. Although, the prevalence of chronic HBV infection varies geographically: in sub-Saharan Africa, the seroprevalence of HBs antigen (HBsAg) in the adult population remains high, over 8% [2] in Senegal, 85% of population have at least one hepatitis B virus (HBV) marker and the prevalence of HBsAg varies between 11 to 17% [3]. Without appropriate clinical and biological management, about 5% to 10% of the HBsAg carriers will progress into chronic hepatitis B and finally into hepatic cirrhosis and liver cancer, while a few patients may develop a life threatening acute liver failure for acute hepatitis B (AHB) [4]. Also, viral DNA detection is not recommended for HBV infection diagnosis, HBV DNA quantification is a key point for an accurate follow-up of chronic HBV carriers and, particularly when evaluating patient response to treatment. The indication of antiviral treatment in patients with chronic hepatitis is, according to European association for the study of the liver (EASL) recommendations, based on the combination of three major criteria: serum level of viral DNA, serum levels of transaminases, and liver histology. Viral load monitoring is also required at the start of treatment (after 8-12 weeks) to assess the primary response, and then at long-term intervals (every 3-6 months) is needed to evaluate treatment efficacy and detect the likely occurrence of treatment drug resistance. Real time PCR is considered as a gold standard method for quantification of HBV viral load [5]. In Senegal two HBV DNA assay (COBAS AmpliPreP/COBAS Taqman® Roche molecular diagnostics, Basel Switzerland), and m2000 (Abbott molecular diagnostics, Wiesbaden, Germany), are frequently used for the quantification of HBV DNA. Recently, a new real-time PCR technique Amplix NG 16 (Biosynex, Strasbourg France) has been introduced in the country. With the high cost of Cobas and m2000 tests, long time of performing these tests with these PCR machine and the need to have laboratories with certain technical level, it is important to evaluate it´s performance on site, and with the local population, as recommended. The objective of this study was to evaluate the RT PCR method (Amplix) performance used in the laboratory of Joint Military Medical center to compare the results obtained with those COBAS AmpliPrep/COBAS TaqMan.

## Methods

**Patients and samples:** this study included patients with chronic HBV followed up for evaluation viral HBV replication and by health service of gastrology in Senegal. The study included patients with chronic HBV followed for assessment of viral HBV replication and the effectiveness of antiretroviral treatment in some gastroenterology departments in Senegal. From each patient, a total of 5ml of blood samples were collected by venipuncture using ethylene diamine tetra acetic (EDTA) tubes. Cells separation was processed to obtain plasma within 30 minutes after collection. The plasma samples were then divided into two aliquots, numbered sequentially, and stored at-20°C at the laboratory of medical center joint of army until testing (i.e. from February to June 2016). Hepatitis B virus DNA quantification assays. Study was performed in two steps: the first step was to verify on site the HBV Amplix® real time polymerase chain reaction (RT PCR) performances (repeatability, reproducibility, accuracy and uncertainties) using two controls level (4 and 7 log IU/ml of viral load) and the second step was to compare viral load quantitation using COBAS AmpliPrep/COBAS Taqman HBV assay (CAP/CTM v2.0). Viral quantitation was performed for each platform according to the manufacturer´s instructions.

Hepatitis B virus DNA quantification Amplix® Assay is an automated real-time PCR based on the amplification of a specific conservative DNA sequence of an open reading frame X (ORFx) and fluorescent detection of amplified DNA in real-time mode. The HBV DNA preparation with Amplix NG (new generation) 48 instrument requires 50μL of plasma. One Template DNA was extracted from 200μl of plasma and 5μl of International Standard with an automatic ExiPrep Dx viral DNA/RNA kit on an Amplix NG 16. DNA is extracted and eluted in a volume of 50μL. After extraction, 30μl of master mix and 10μl of DNA template are mixed into tubes of 0.2ml. The master mix contains reagents and enzymes for specific amplification of a region of the HBV X gene and for direct detection of the amplicon. An internal standard (IS) included in the reaction mix, controlling the possible inhibition of the PCR reaction and the efficiency of the DNA isolation process. Amplification was performed using Amplix NG 48. The PCR program was: 37°C for 2min, 95°C for 10min for 1 cycle (first denaturation), 95°C for 5s (second denaturation), 60°C for 40s for 45 cycle (hybridation), and 72°C for 20s (polymerisation) for 45 cycle. Hepatitis B virus DNA levels were expressed in international units per milliliter (IU/mL) with a conversion factor of 4.20 copies of HBV DNA per IU. The lower limit of detection (LOD) provided by the manufacturer is 26.0 IU/mL, and the dynamic range of quantification is from 26.0 to 1.3 108 IU/mL (1.41-8.74 log10 IU/mL) (Table 1).

**Table 1:** characteristics of two Hepatitis B virus (HBV) DNA quantification assays

	Assays	
Variables	CAP/CTM v2.0	Amplix
DNA extraction principle	Magnetic particle	Magnetic particle
Sample volume (μL)	650 μl	200 μl
Elution volume (μL)	65 μl	50 μl
Sample capacity per batch	4-72	1-48
Extraction runtime (min)	2h 15 mn	1h 30 mn
Amplification runtime (min)	3h 30 mn	1h 40 mn
Target HBV genome region	Precore and Core	X
Claimed low limit of detection (IU/mL)	20	26
Claimed dynamic range (IU/mL)	2.00x10^1^ to 1.70x10^8^	2.6 0x10^1^ to 1.3x10^8^

COBAS AmpliPrep/COBAS Taqman assay allows the automated preparation of samples followed by automated amplification and detection of target HBV DNA and an internal quantitation standard. The reagent master mix contains primer pairs and probes specific to HBV DNA and the internal quantitation standard. Detection of amplified DNA is conducted by means of a double-labelled oligonucleotide probe specific to the target and the internal quantitation standard, which allows independent amplicon identification of both targets. The measurement range of the CAP/CTM assay is 20.0 to 1.7x108 IU/mL (1.3-8.2 log10 IU/mL) (Table 1). For the determination of the contamination, the 7log10 IU/ml control was analyzed three successive times (H1, H2, H3 with mH on average) followed by the 4 log10 IU/ml control also analyzed three times in a row (B1, B2, B3). The sequence H1, H2, H3 and B1, B2, B3 was repeated 5 times to determine the average B1 (mB1) and B3 (mB3). A statistically significant difference between the two means was established with the Student test before calculating the percentage of contamination according to the following formula:

$$\%C=\frac{\left(mB1-mB3\right)}{\left(mB-mB3\right)}\times 100$$

mB1: low control average, mH: hight control average and C = contamination. Statistical analysis was performed using Excel (MS), Epi info V.6 and method validator software. Pearson´s correlation coefficients and linear regression were used for correlation analysis. Bland-Altman tests were used to assess the agreement of the quantitative results of HBV DNA between Amplix and CAP/CTM assay. Differences between viral loads were considered as significant when the value was greater than 0.5log IU/ml and P=0.05.

## Results

**Agreement and correlation between the two assays:** among the 42 serum samples tested, HBV DNA was quantified in 35 samples (83.3%, CI95%= 68.6-93) by Amplix assay, and 34 (81.0%, CI95%=65.9-91.4) were quantified by the CAP/CTM. The detection rate by the CAP/CTM assay was significantly lower than that by the Amplix assay (P <0.02). The odd ratio between the qualitative results of the two assays was 10.39 (CI95%=1.67 to 64). Hepatitis B virus viral load was detected in 38 (90.8%) samples and not detected in 4 (9.52%) samples out of 42 (Table 2). However, Amplix assay detected HBV viral load in 4 (11.4%) samples not detected by CAP/CTM. Conversely, the CAP/CTM assay detected 3 (8.8%) positive samples but not Amplix. The mean ± SD of HBV DNA was 4.33±2.15 log IU/mL for Amplix assay and 3.93±2.01 log IU/mL for the CAP/CTM v2.0 assay. The viral loads quantified by Amplix assay were significantly higher than those quantified by the CAP/CTM assay (P=0.0001). Further, 19 (45.23%) samples showed significant difference of a load 0.5 log IU/mL (Table 3).

**Table 2:** comparison and difference of quantification results of the HBV DNA between Amplix and CAP/CTM technical

	Amplix					
**CAP/CTM**		**No detected**	**[1.42 - 3.30]**	**[3.30-4.30]**	**≥ 4.30**	**Total**
	**No detected**	4	4	0	0	8
	**[1.30-3.30]**	3	11	3	1	18
	**[3.30 - 4.30]**	0	0	0	3	3
	**≥ 4.30**	0	0	1	12	13
	**Total**	7	15	4	16	42

**Table 3:** difference of quantification results of the HBV DNA between Amplix and CAP/CTM technical

Log10 IU /ml viral load difference				
**Viral load (Log10 IU/ml)**	Δ ≤ 0.5	0.5< Δ≤1	Δ >1	Total
< 3,3	9 (64.3%)	4 (28.6%)	1(7.1%)	14
[3,3 - 4,30]	1 (33.1 %)	1 (33.1%)	1 (33.3%)	3
[4,30 - 5,30]	2 (28.6%)	4 (57.1%)	1(14.3)	7
> 5,30	3 (30%)	4 (40%)	3 (30%)	10

**Figure 1:**
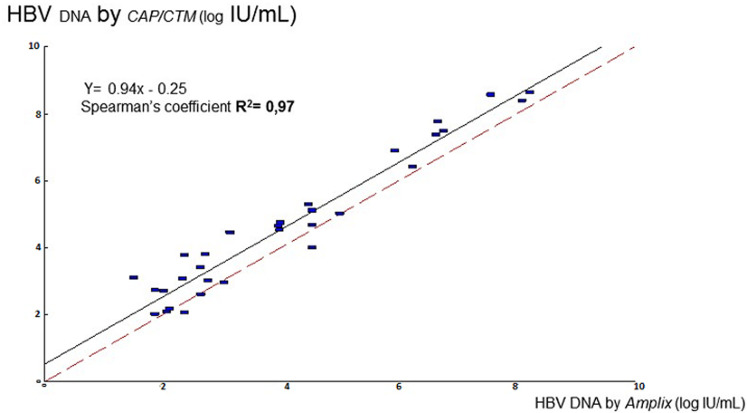
the Passing-Bablok equation

**Figure 2:**
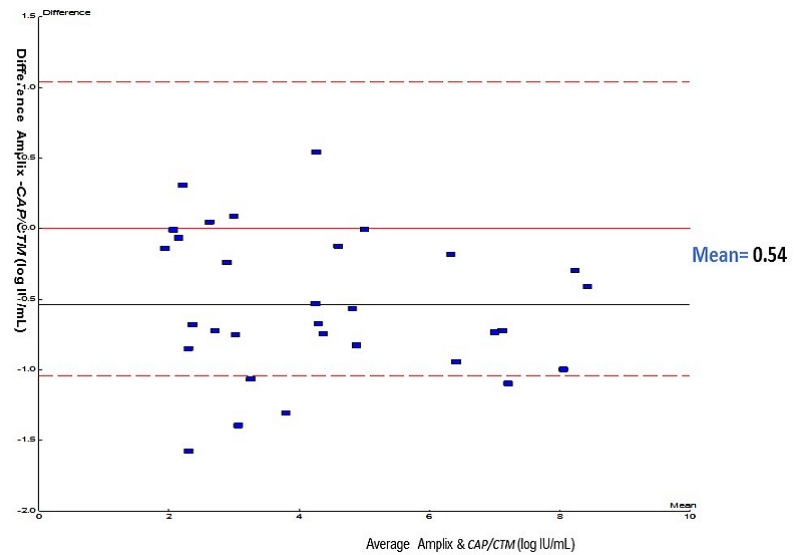
the mean difference (Amplix - CAP/CTM) in Bland-Altman plot

**Linear regression analysis:** linear regression analysis between Amplix with CAP/CTM assays was carried out for the viral load quantified by these assays (Figure 1). Comparison of Amplix with CAP/CTM showed strong correlation between these assays (r^2^=0.97, p=0.001). Level of agreement. A Bland-Altman plot was used to determine the agreement between Amplix with CAP/CTM assays. Using this method, the differences between the HBV DNA log IU/ml values of two assays were plotted against the averages of the two techniques. The mean difference between Amplix with CAP/CTM was 0.54 log10 IU/ml (CI=0.37-0.71) with limits of agreement of-0.54 to 1.57 log1 IU/ml (Figure 2). The repeatability of the technique was evaluated by testing 2 concentrations levels (4 log IU/ml and 7 log IU/ml). These two concentrations were tested 15 times each in the same manipulation. Catalogue value (CV) obtained were 2.12% and 1.60% for 4 log IU/ml and 7 log IU/ml respectively (Table 4). Intermediate fidelity (reproducibility). The intra-laboratory reproducibility of the technique was evaluated by testing the 2 concentrations levels of 4 log IU/ml and 7 log IU/ml. The CV obtained were 6.65 % and 3.15%, respectively, for these two concentrations.

**Table 4:** performance criteria of Amplix Assay

	Mean (M)	4.86
Repeatability (4 log IU/ml)	Standard deviation (ET)	0.09
	CV (%)	2.12
	Mean (M)	7.76
Repeatability (7 log IU/ml)	Standard deviation (ET)	0.12
	CV (%)	1.60
	Mean (M)	3.91
Reproducibility (4 log IU/ml)	Standard deviation (ET)	0.26
	CV (%)	6.65
	Bias	-2.10
	Accuracy	-0.08
	Uncertainty	0.92
	Mean (M)	7.22
Reproducibility (7 log IU/ml)	Standard deviation (ET)	0.23
	CV (%)	3.15
	Bias	3.20
	Accuracy	0.22
	Uncertainty	0.98

CV: coefficient of variation

**Figure 3:**
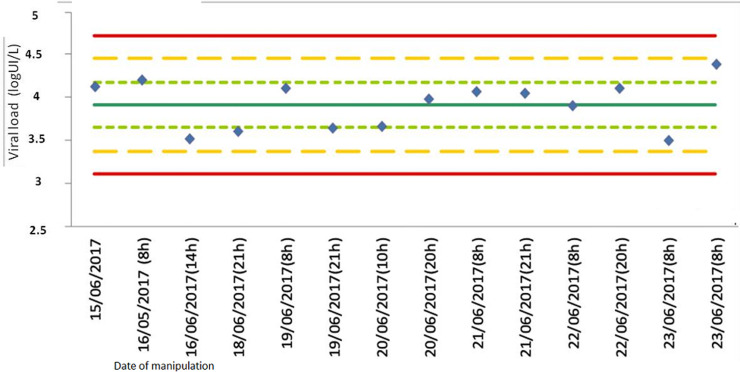
diagram of levey-jennings of concentration level 4 log10 IU/ml

**Figure 4:**
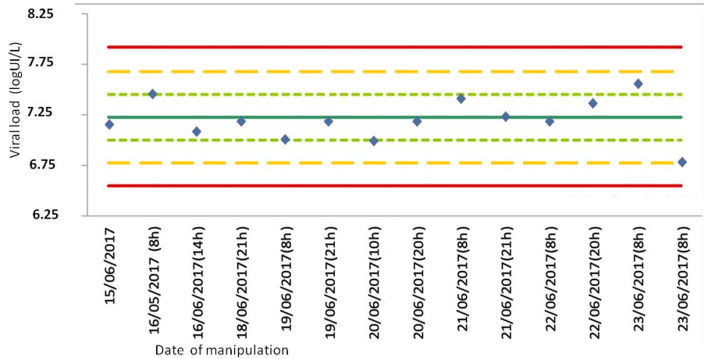
diagram of levey-jennings of concentration level 7 log10 IU/ml

The CV obtained for the 2 levels are low, so the dispersion of the values around the mean is low. To confirm the results of the intermediate fidelity, we traced the control diagram used, Levey-Jennings (Figure 3, Figure 4). The diagram made to represent the different point of the concentrations levels according to their dispersion around the mean, as function of time. The concentration level (4 log IU/ml) are between M±ET for 60%. The remaining 30% does not exceed M±2 (Figure 3). Eighty percent 80% of the values of the concentration level equal to 7 log IU/ml do not exceed M±1ET, 20% of the values are greater than M±1ET without exceeding M±2ET (Figure 4). Accuracy and uncertainties. Accuracy and uncertainties were evaluated based on the results of reproducibility of the two panels (4 and 7 log10 IU/ml). For accuracy, the CV values were 0.08% for 4 log10 IU/ml and 0.22 % for 7 log10 IU/ml. The uncertainties were ±0.53 for 4 and 7 log10 IU/ml, each. The level of contamination evaluated 5 times using 2 values (7 and 4 log10 IU/ml) was 2.88%.

## Discussion

Chronic hepatitis B (CHB) is a major cause of cirrhosis and hepatocellular carcinoma in Southeast Asia, China, and Africa [6]. Hepatitis B virus DNA quantification is essential for monitoring disease status and treatment response in CHB patients. Until now, several commercially available real-time PCR assays for this have been developed. Newly Amplix® real-time PCR for HBV assay suggested a higher Limit of detection (LOD), lower sample volume, and testing than CAP/CTM by manufacturer. No studies have yet evaluated this new PCR assay in the quantification of the HBV genome. In our study, there were significant differences between the CAP/CTM v2.0 and Amplix® in the detection rate and viral load when quantifying HBV DNA levels in serum samples, even though good correlation was observed (r= 0.97). The HBV DNA levels determined by Amplix assay were substantially higher than the results by the CAP/CTM assay. According to the manufacturer´s protocol, the LOD of Amplix assay (26 IU/mL) is higher than that of the CAP/CTM assay (20 IU/mL). However, our results from Amplix assay showed a slightly higher positive rate than those from the CAP/CTM.

There are several possible factors that may lead to assay discrepancies. Mutations in precore and core promoter regions may occur as CHB progresses [7]. These mutations may influence the result of HBV DNA quantification concluded [8] that the detection of viral load samples may be influenced by the tyrosine-methionine-aspartate-aspartate (YMDD) mutation, which confers lamivudine resistance. In Senegal the most predominat genotype are A and E. In addition, a study carried out in Japan showed that that the B genotype and low HBV viral load were two factors that contribute to significant differences in HBV DNA viral load detection [9]. We also evaluated the performance of HBV DNA measurement by the HBV DNA-based Amplix real-time PCR assay developed by biosynex. The analytical performance characteristics of Amplix were correlated to other RT-PCR assays. The detection threshold of 26 UI/ml was close to the threshold generally reached with other commercial assays. The linearity range from 26 UI/ml to 1.3x108 UI/ml allows only one test in case of high viral load. Amplix was highly reproducibility with good intra-and-interrum below 15% as recommended for nucleic acid-based technologies [10]. The Amplix assay was highly accurate with an average bias below 5%. The main limitation of the current study included a relatively small sample size.

## Conclusion

Amplix® real-time PCR method has proven to be satisfactory in terms of efficiency, repeatability, reproducibility and accuracy. Good correlation and agreement with the Cobas Taqman™ technique was also noted. In addition, the test series are feasible with a smaller number of samples and are performed with a shorter turn around time than conventional techniques. Thus inter change ability between these two techniques can be accepted and the Amplix® real-time PCR platform considered a reliable system for monitoring patients with chronic hepatitis B.

### What is known about this topic

- The viral load of hepatitis marker essential for monitoring Hepatitis B virus is poorly performed in routine laboratories.

### What this study adds

- Feasibility of viral load in decentralized areas.
